# Adipokine Chemerin Stimulates Progression of Atherosclerosis in ApoE^−/−^ Mice

**DOI:** 10.1155/2019/7157865

**Published:** 2019-10-31

**Authors:** Huadong Liu, Wei Xiong, Yu Luo, Hua Chen, Yaqiong He, Yuanzhi Cao, Shaohong Dong

**Affiliations:** ^1^Department of Cardiology, Shenzhen People's Hospital, The Second Affiliated Hospital of Jinan Medical College, Shenzhen 518020, China; ^2^Department of Gerontology, Shenzhen People's Hospital, The Second Affiliated Hospital of Jinan Medical College, Shenzhen 518020, China; ^3^Department of Emergency, Shenzhen People's Hospital, The Second Affiliated Hospital of Jinan Medical College, Shenzhen 518020, China

## Abstract

**Background:**

Vascular remodeling is the most critical pathogenesis of atherosclerosis. Adipokine chemerin was known for its relationship with obesity as well as metabolism. Most recently, chemerin was found to play a crucial role in the pathologic process of cardiovascular diseases including coronary heart disease. In this study, we surveyed the role of chemerin in progression of atherosclerosis in ApoE^−/−^ mice.

**Objective:**

To investigate the relationship between chemerin and progression of atherosclerosis in ApoE^−/−^ mice and its mechanism.

**Methods:**

8-week-old ApoE^−/−^ mice were fed with high-fat diet to induce the atherosclerosis model. Adenoviruses were transfected for knockdown or overexpression of chemerin gene into aorta. Serums and aortic tissues of ApoE^−/−^ mice were obtained after feeding high-fat diet for 16 weeks. HE staining and oil red staining were performed to evaluate aortic plaque. ELISA was performed to explore serum levels of tumor necrosis factor-*α* (TNF-*α*), interleukin-1*β* (IL-1*β*), and transforming growth factor-*β*1 (TGF-*β*1). Real-time PCR and western blotting were carried out to investigate the mRNA and protein levels of chemerin, nuclear factor-*κ*B p65 (NF-*κ*Bp65), proliferating cell nuclear antigen (PCNA), phosphorylated p38 mitogen-activated protein kinase (*p*-p38-MAPK), phosphorylated c-Jun N-terminal kinase (*p*-JNK), and phosphorylated extracellular signal regulated kinase 1/2 (*p*-ERK 1/2).

**Result:**

Aortic plaque formation was significantly induced by high-fat diet in ApoE^−/−^ mice. Simultaneously, elevated serum levels of TNF-*α* and IL-1*β* and elevated mRNA and protein levels of chemerin, NF-*κ*Bp65, PCNA, *p*-p38-MAPK, *p*-JNK, and *p*-ERK 1/2 were found in ApoE^−/−^ mice. After aortic chemerin gene was inhibited by adenovirus, aortic atherosclerosis induced by high-fat diet was significantly meliorated, serum levels of TNF-*α* and IL-1*β* decreased, mRNA and protein levels of NF-*κ*Bp65, PCNA, *p*-p38-MAPK, *p*-JNK, and *p*-ERK 1/2 decreased simultaneously.

**Conclusion:**

Our study revealed that chemerin stimulated the progression of atherosclerosis in ApoE^−/−^ mice.

## 1. Introduction

Coronary heart disease and stroke originated from atherosclerosis are the primary causes of death worldwide [1, 2]. Despite great efforts have been made, the pathogenic molecular mechanisms of atherosclerosis is not clearly clarified. It was extensively agreed that atherosclerosis is a chronic progressive inflammatory and immune disorder [3–6]. Adipokines are cytokines secreted by adipose tissue, which play a critical role in adipocyte differentiation and maturation. Under pathological states such as tissue damage and inflammation, large amounts of adipokines are synthesized and secreted, which are not only associated with metabolic diseases such as obesity and type 2 diabetes but also contribute to the development of cardiovascular diseases such as coronary heart disease [7]. Chemerin, a latest discovered adipokine, was found to play an important role in cardiovascular diseases [8]. Evidences showed that circulating levels of chemerin significantly increased in rheumatoid arthritis patients accompanied with cardiovascular disease, gestational hypertension, metabolic syndrome, dilated cardiomyopathy, and advanced carotid stenosis [9–13]. It also was identified by autopsy that chemerin was expressed in adipose tissue around the human coronary arteries, vascular smooth muscle cells (VSMCs), and foam cells in coronary artery lesions, the level of which was positively correlated with the severity of coronary atherosclerosis [14]. However, it is less known about the mode and mechanism of chemerin involving in the cardiovascular diseases including atherosclerosis and its complications.

Vascular remodeling is the crucial pathological process in atherosclerosis and restenosis after angioplasty. VSMCs and endothelial cells are primary cell types in blood vessel walls, which are fundamental to vascular homeostasis [15, 16]. In a previous study, we found that circulating chemerin levels increased after carotid balloon injury, and knockdown of chemerin significantly inhibited the VSMCs proliferation in vitro as well as prohibited carotid neointimal hyperplasia in vivo after angioplasty [17]. Further investigation revealed that knockdown of chemerin receptor, chemokine-like receptor 1 (CMKLR1), inhibited the proliferation and migration of VSMCs [18].

In the present study, we explored the role of chemerin in initiation and progression of atherosclerosis in apolipoprotein-E deficient (ApoE^−/−^) mice to better understand its duty and the underlying mechanism in atherosclerosis and other cardiovascular diseases.

## 2. Materials and Methods

### 2.1. Ethics Statement

This study was strictly performed according to the recommendations in the Guide for the Care and Use of Laboratory Animals of the National Institutes of Health. The protocol was sanctioned by the Animal Ethics Committee of the Shenzhen People's Hospital. All efforts have been made to reduce animal suffering.

### 2.2. Adenovirus Construction

As described in our previous study [19], the synthesized chemerin gene DNA was digested by PestI and HindIII to generate the target sequence, which was integrated into the adenoviral vector pDC316-mCMV-ZsGreen. Simultaneously, the synthetic shRNA interfering chemerin gene was digested by Pst I and BamH with the pDC316-ZsGreen-shRNA vector. Chemerin shRNA sequence, forward: GGCACAATCAAACCAAACGGGATCAAGAGTCCCGTTTGGTTTGATTGTGCTTTTTTG, reverse: GATCCAAAAAAGCACAATCAAACCAAACGGGACTCTTGATCCCGTTTGGTTTGATTGTGCCTGCA. The digested product was harvested, and the shRNA was cloned into the pDC316-ZsGreen-shRNA vector. Two above recombinant plasmids were transfected, respectively; subsequently, the positive recombinant plasmids were picked and identified. The recombinant adenovirus plasmid overexpression of chemerin gene as well as knockdown of chemerin gene were transfected into the 293a cells, respectively, with the adenovirus packaging plasmid to generate the recombinant adenoviruses. The adenoviruses were repeatedly harvested and purified to produce the efficient chemerin gene overepression adenovirus (overexpression) as well as chemerin gene interference adenovirus (knockdown) for further experiments.

### 2.3. Mice and Diet

7-week-old male Apoe^−/−^ mice and wild-type C57BL/6J mice were obtained from Vital River Laboratory Animal Technology Co., Ltd (Beijing, China) and housed in specific pathogen-free animal rooms in climate-controlled conditions (22 ± 1°C, 40%–70% humidity) as well as diurnal conditions (12 hour light/dark cycle) with access to food and water ad libitum. After acclimatization for 1 week, 25 ApoE^−/−^ mice were fed high fat diet (40 kcal% fat, 1.25% cholesterol, Research Diets D12108C, USA) and, respectively, sacrificed at 0, 4, 8, 12, and 16 weeks (*n* = 5 for each group). The wild-type C57BL/6J were fed with low-fat diet (10 kcal% fat, 0% cholesterol, Research Diets D12102C, USA) for 16 weeks (control group, *n* = 6). Another 36 ApoE^−/−^ mice were fed high fat diet for 16 weeks. In the 8th week, the ApoE^−/−^ mice were injected with the vector (atherosclerosis + vector group, *n* = 6), the chemerin gene overexpression adenovirus (Atherosclerosis + Overexpression group, *n* = 6), or chemerin gene knockdown adenovirus (Atherosclerosis + Knockdown group, *n* = 6), respectively, from caudal vein every 2 weeks until the 16th weeks. The mice of the control group were injected with same volume of PBS in the same manner.

### 2.4. Aortic Morphology

The thoracic aortas were isolated. These samples were embedded in optimum cutting temperature (OCT) compound (Sakura Fine Technical, Tokyo, Japan) and snap-frozen in liquid nitrogen. OCT-embedded samples were cut serially into cross-sections (5 *μ*m thick). Hematoxylin-eosin staining and oil red O staining were performed to evaluate aortic morphology, respectively. The thickness of neointima and media, lipid area, and plaque area were investigated by calculating the neointima/media ratio and lipid percentage using computer-assisted color image analysis (Image-Pro Plus, version 6.0, Media Cybernetics, Inc., Silver Spring, Maryland, USA).

### 2.5. Immunofluorescence Assay

Thoracic aortic tissues isolated at different stages were fixed in 4% paraformaldehyde and permeabilized using 0.1% Triton X-100 at room temperature and then blocked by 5% BSA. Slides were incubated with polyclonal rabbit anti-rat chemerin (1 : 1000, Abcam, UK). Subsequently, tissues were treated with goat anti-rabbit immunoglobulin G (1 : 500, Invitrogen Life Technologies, USA) and stained by 4′,6-diamidino-2-phenylindole (DAPI). The confocal microscope (Leica, Germany) was employed to capture fluorescence images. All parameters were measured by computer-assisted color image analysis.

### 2.6. Enzyme-Linked Immunosorbent Assay

All blood samples from mice were collected after feeding low-fat diet or high-fat diet for 16 weeks. Serum levels of interleukin-1*β* (IL-1*β*), tumor necrosis factor-*α* (TNF-*α*), and transforming growth factor-*β*1 (TGF-*β*1) were assessed using an enzyme-linked immunosorbent assay (ELISA) kit according to the manufacturer's protocol (4A Biotech Co., Ltd, China).

### 2.7. Real-Time PCR

Total RNA was extracted from thoracic aortas and reversed to cDNA using the Transcript cDNA Synthesis Supermix kit (Takara, Japan). Quantitative real-time PCR was performed to assess chemerin, nuclear factor-*κ*B p65 (NF-*κ*Bp65), proliferating cell nuclear antigen (PCNA), phosphorylated p38 mitogen-activated protein kinase (*p*-p38-MAPK), phosphorylated c-Jun N-terminal kinase (*p*-JNK), and phosphorylated extracellularsignal regulated kinase 1/2 (*p*-ERK 1/2) using the ABI PRISM 7900 Sequence Detection System (Applied Biosystems, USA) with the Top Green qPCR Supermix kit (Takara, Japan). U6 RNA served as an internal control. Primers were as follows: chemerin forward (5′ to 3′) TACAGGTGGCTCTGGAGGAGTTC, chemerin reverse CTTCTCCCGTTTGGTTTGATTG; NF-*κ*Bp65 forward (5′ to 3′) CACGAGATGCCATCCTGGAC, NF-*κ*Bp65 reverse (5′ to 3′) AAGGCCTCAAGGAACAAGT; PCNA forward (5′ to 3′) TTTGAGGCACGCCTGATCC, PCNA reverse (5′ to 3′) GGAGACGTGAGACGAGTCCAT; *p*-JNK forward (5′ to 3′) GGAATCAAGCACCTTCACTCTG, *p*-JNK reverse (5′ to 3′) AGTCACCACATAAGGCGTCA TC; *p*-ERK 1/2 forward (5′ to 3′) CTGGCTITCTGACCGAGTATGTG, *p*-ERK 1/2 reverse (5′ to 3′) CAATITAGGTCCTCTYGGGATG; *β*-actin forward (5′ to 3′) AGCGGTTCCGATGCCCT, *β*-actin reverse (5′ to 3′) AGAGGTCTTTACGGATGTCAACG.

All primers were synthesized by Sangon (China). The relative expression levels of mRNA were calculated based on the following equation: relative mRNA level = 2^−(ΔCt sample−ΔCt control)^. All operations were carried out according to the manufacturer's instructions.

### 2.8. Western Blotting

Total proteins (20 *μ*g) extracted from thoracic aortas were resolved on 10% SDS-polyacrylamide gels and electrotransferred onto nitrocellulose membranes by electrophoresis. The membranes were blocked in 5% nonfat milk and TBS containing 0.3% Tween-20, subsequently incubated overnight with polyclonal rabbit anti-rat chemerin (1 : 1000, Abcam, UK), NF-*κ*Bp65 (1 : 1000, Cell Signaling Technology, USA), PCNA (1 : 500, Santa Cruz, USA), *p*-p38-MAPK (1 : 1000, Santa Cruz, USA), *p*-ERK 1/2 (1 : 1000, Santa Cruz, USA), *p*-JNK (1 : 500, Cell Signaling Technology, USA), and *β*-actin (1 : 1000, Santa Cruz, USA). The blots were incubated in 1 : 6000 dilution of goat anti-rabbit horseradish peroxidase-conjugated secondary antibodies. 100 *μ*l ECL solution was added in each sample to detect the protein signal with the Quant RT ECL cold CCD imaging system (General Electric, USA).

### 2.9. Statistical Analysis

The number of animals was determined based on the previous study [17]. Statistical analyses were performed using SPSS 12.0 (SPSS Inc., USA). The normality of data was first assessed by Shapiro–Wilk testing. The data that obeyed a normal distribution were expressed as mean ± standard error. For relative gene expression, mean value of the control group was defined as 100%. One-way analysis of variance (ANOVA) followed by the Student–Newman–Keuls test were used for statistical analyses. A *p* value <0.05 was considered statistically significantly.

## 3. Results

### 3.1. Chemerin Promoted Aortic Atherosclerosis in ApoE^−/−^ Mice

**Figure 1:**
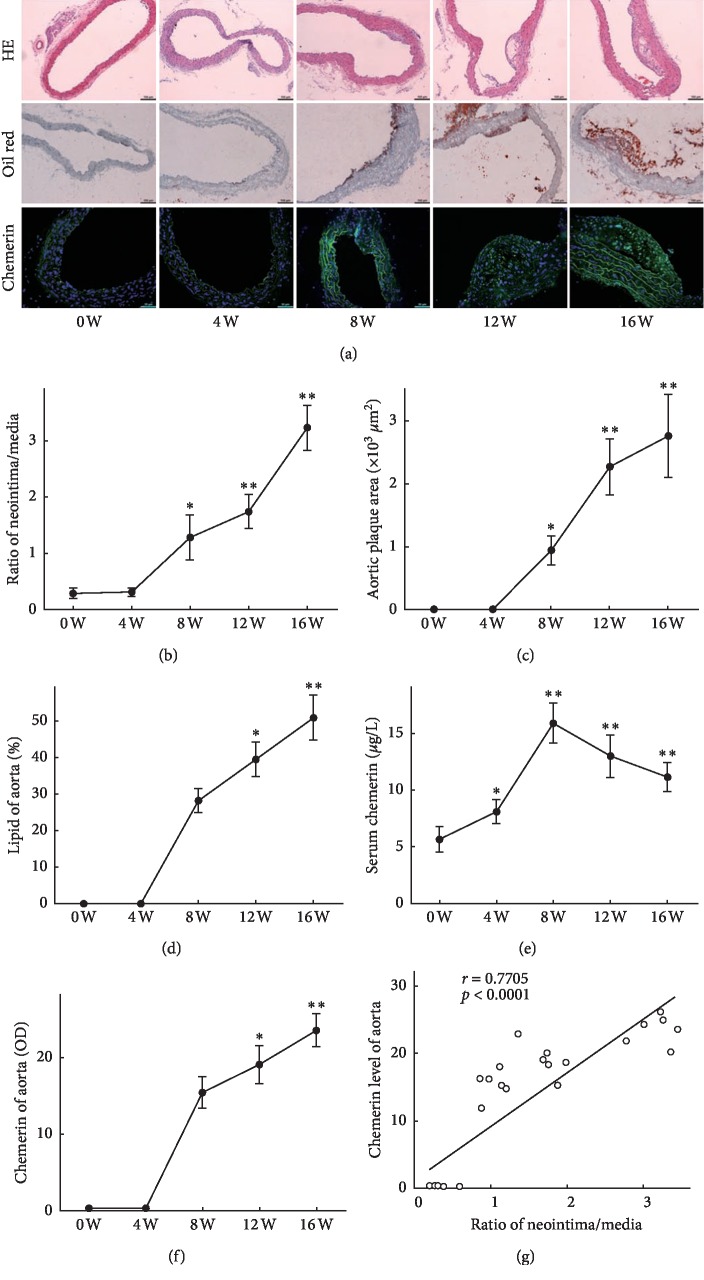
The circulating and aortic levels of chemerin increased in the progression of aortic atherosclerosis in ApoE^−/−^ mice. (a) The aortic morphology staining by HE, oil red O, and immunofluorescence assay for chemerin at different stages (0, 4, 8, 12, and 16 W) in ApoE^−/−^ mice fed with high-fat diet. (b), (c), and (d) The ratios of aortic neointima/media thickness, aortic plaque areas, and lipid percentages of aortic tissue at different stages. (e) Serum levels of chemerin investigated by ELISA at different stages. (f) The expression of chemerin in aortic tissues investigated by immunofluorescence assay at different stages. (g) Correlation analysis between aortic chemerin level and the ratio of aortic intima/media thickness. Scale bar: 0.1 mm. ^*∗*^*P* < 0.05, ^*∗∗*^*P* < 0.01 compared with 0 W.

After being administrated with high-fat diet for 8 weeks, lipid stripes were initially found in the aortic tissue of ApoE^−/−^ mice (Figure 1(a)). The ratio of aortic neointima/media thickness and aortic plaque area at the 8th week were significantly elevated (*vs* 0 week, *P* < 0.05. Figures 1(b) and 1(c)), the lipid percentage of aortic tissue increased at the 12th week (*vs* 0 week, *P* < 0.05.  Figure 1(d)). The most significant atherosclerotic plaques were found at the 16th week (Figure 1(a)). Compared with the level of ApoE^−/−^ mice in 0 week, serum chemerin started to increase at the 4th week (*P* < 0.05), peaked at the 8th week (*P* < 0.01), and decreased at the 12th week (*P* < 0.01) (Figure 1(e)). Immunofluorescence assay showed that positive staining of chemerin (green fluorescence) was primarily located in the cytoplasm of VSMCs (Figure 1(a)). The positive expression of chemerin in the aortic tissue of ApoE^−/−^ mice was not obvious in the first 8 weeks, which significantly increased at the 12th week (*vs* 0 week, *P* < 0.05) and peaked at the 16th week (*vs* 0 week, *P* < 0.01) (Figure 1(f)). A positive correlation was found between aortic chemerin level and the ratio of aortic intima/media thickness (Figure 1(g)).

**Figure 2:**
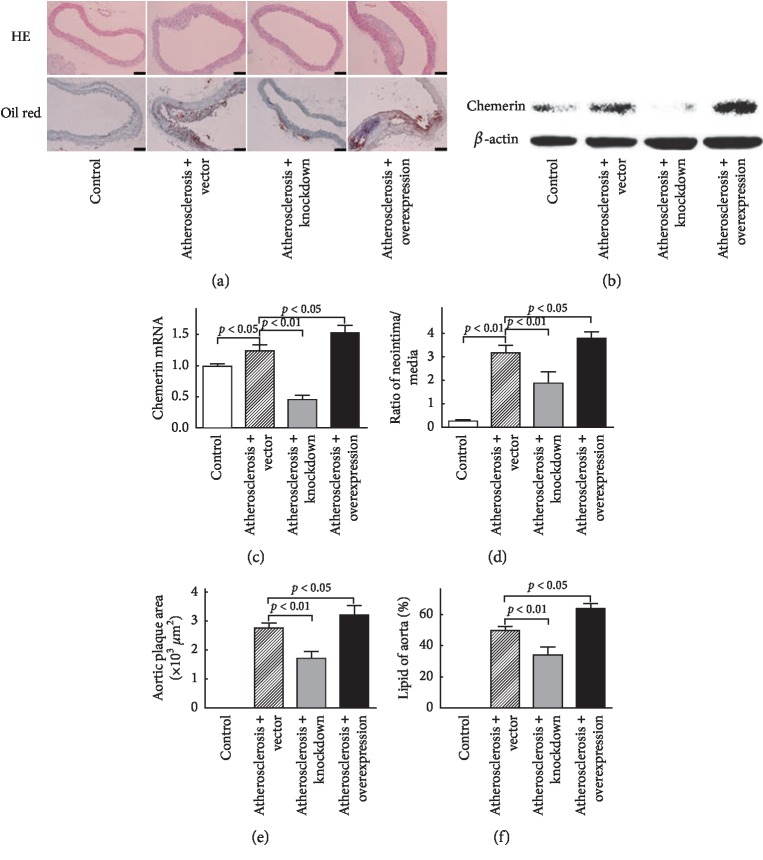
Chemerin stimulated the aortic atherosclerosis in ApoE^−/−^ mice. (a) The aortic morphology stained by HE and oil red in ApoE^−/−^ mice fed with high-fat diet and transfected with adenovirus. (b) The mRNA level of aortic chemerin. (c) The protein level of aortic chemerin. (c–e) The ratios of aortic neointima/media thickness, aortic plaque areas, and lipid percentages of aortic tissue. Scale bar: 0.1 mm.

In the Atherosclerosis + Vector group, the mRNA and protein levels of aortic chemerin were significantly increased (*vs* control group, *P* < 0.05. Figures 2(b) and 2(c)). Compared with the Atherosclerosis + Vector group, the expression of chemerin in aortic tissues was decreased in the Atherosclerosis + Knockdown group (*P* < 0.01, Figure 2(b), 2(c)) and increased in the Atherosclerosis + Overexpression group (*P* < 0.05, Figures 2(b) and 2(c)). As shown in Figures 2(a), 2(d), and 2(f), the atherosclerotic plaque formation was significantly reduced in the Atherosclerosis + Knockdown group and augmented in the Atherosclerosis + Overexpression group, as compared with the Atherosclerosis + Vector group.

### 3.2. Chemerin Increased Serum Proinflammatory Cytokine Levels in ApoE^−/−^ Mice

**Figure 3:**
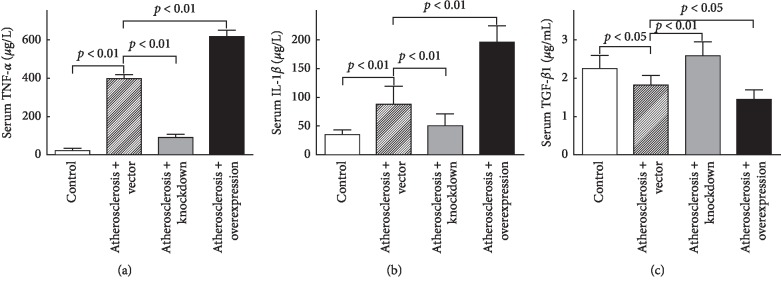
Chemerin increased the serum proinflammatory cytokines levels in ApoE^−/−^ mice. (a, b) The serum levels of TNF-*α* and IL-1*β* were significantly raised by chemerin. (c) The serum level of TGF-*β*1 was significantly decreased by chemerin.

In the atherosclerosis + vector group, serum levels of TNF-*α* (Figure 3(a)) and IL-1*β* (Figure 3(b)) significantly increased (*P* < 0.01), whereas the serum level of TGF-*β*1 (Figure 3(c)) significantly decreased (*P* < 0.05), as compared with the control group. In the Atherosclerosis + Knockdown group, serum levels of TNF-*α* (Figure 3(a)) and IL-1*β* (Figure 3(b)) significantly decreased (*P* < 0.01), whereas the serum level of TGF-*β*1 (Figure 3(c)) significantly increased (*P* < 0.01), as compared with the Atherosclerosis + Vector group. In the Atherosclerosis + Overexpression group, serum levels of TNF-*α* (Figure 3(a)) and IL-1*β* (Figure 3(b)) significantly increased (*P* < 0.01), whereas serum level of TGF-*β*1 (Figure 3(c)) significantly decreased (*P* < 0.05), as compared with the Atherosclerosis + Vector group.

Chemerin promoted the phosphorylation of p38 MAPK and expression of NF-*κ*Bp65.

**Figure 4:**
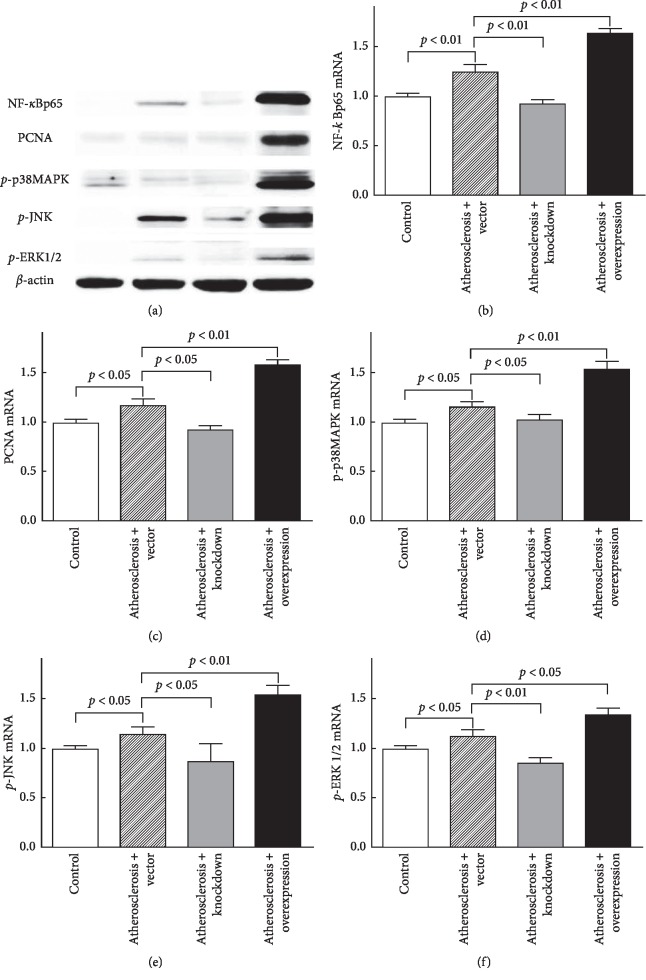
Chemerin promoted the phosphorylation of p38 MAPK and expression of NF-*κ*Bp65. (a) The protein levels of NF-*κ*Bp65, PCNA, p-p38MAPK, *p*-JNK, and p-ERK1/2. (b–f) The mRNA levels of NF-*κ*Bp65, PCNA, p-p38MAPK, *p*-JNK, and p-ERK1/2.

In the Atherosclerosis + Vector group, the mRNA and protein expressions of NF-*κ*Bp65 (Figures 4(a) and 4(b)), PCNA (Figures 4(a) and 4(c)), *p*-p38 MAPK (Figures 4(a) and 4(d)), *p*-JNK (Figures 4(a) and 4(e)), and *p*-ERK1/2 (Figures 4(a) and 4(f)) were significantly increased, as compared with the control group. Compared with the Atherosclerosis + Vector group, the mRNA and protein expressions of NF-*κ*Bp65 (Figures 4(a) and 4(b)), PCNA (Figures 4(a) and 4(c)), *p*-p38 MAPK (Figures 4(a) and 4(d)), *p*-JNK (Figures 4(a) and 4(e)), and *p*-ERK1/2 (Figures 4(a) and 4(f)) were significantly decreased in the Atherosclerosis + Knockdown group and increased in the Atherosclerosis + Overexpression group.

## 4. Discussion

Identified in cultured skin cells from the patients with psoriasis, chemerin was originally correlated to maintain the normal physiology of human skin [20]. Further investigation revealed that chemerin is dominantly produced in the liver and white adipose tissue, indicating the role of which as a kind of adipokine [21]. Recent studies have implied that chemerin is closely related to cardiovascular disease. The levels of chemerin were significantly elevated in the synovial fluid of patients with rheumatoid arthritis and could be identified as a biomarker for rheumatoid arthritis [22]. In these patients, increased circulating concentrations of chemerin raised the risk for atherosclerosis [9]. Elevated circulating levels of chemerin were also found in patients with preeclampsia, metabolic syndrome, dilated cardiomyopathy, and carotid stenosis, indicating its role in cardiovascular diseases such as hypertension, coronary heart disease, and cardiac dysfunction [10–13]. In the present study, we found that aortic plaque arose with high-fat diet for 8 weeks, which was most apparent at the 16th week. As same as aortic plaque, chemerin expression was initially found in aortic tissue of ApoE^−/−^ mice at the 8th week, most apparent at the 16th week. A positive relationship was identified between the chemerin level in the aortic tissue and aortic intimal hyperplasia, suggesting that chemerin is involved in atherosclerotic plaque formation in ApoE ^−/−^ mice. Interestingly, the circulating levels of chemerin initially increased at 4th week and peaked at 8th weeks, which were previous to the increasing of aortic chemerin levels. It can be presumed that circulating chemerin was firstly raised due to the obesity and fatty liver induced by high-fat diet feeding, which subsequently led to the increase of chemerin in aortic tissue.

Despite the evidence of anti-inflammation [23], chemerin was extensively acknowledged as a proinflammatory cytokine. It was found that chemein played important roles in regulating immune and inflammatory responses mediated by dendritic cells and natural killer cells [24]. In patients with rheumatoid arthritis, serum levels of chemerin were reduced by anti-TNF therapy, indicating its possible involvement in inflammation [25]. Chemerin promoted the growth and invasion of gastric cancer cells by inducing the phosphorylation of p38 MAPK and the expression of IL-6, vascular endothelial growth factor (VEGF), and matrix metalloproteinase-7 (MMP-7) [26]. The role of chemerin as a proinflammatory cytokine was also demonstrated by the evidence of chemerin-induced MMP production and endothelial angiogenesis [27]. Our present results that elevation of serum TNF-*α* and IL-1*β* induced by high fat diet feeding in ApoE^−/−^ mice were inhibited by knockdown of chemerin supported the opinion that chemerin promotes inflammatory response as a proinflammatory cytokine. However, the relationship between the expression of chemerin in aortic tissue and circulating levels of proinflammatory cytokines including TNF-*α* and IL-1*β* remains unclear.

Three receptors named CMKLR1, G-protein coupled receptor 1 (GPR1), and C-C motif receptor like 2 (CCRL2) have been reported to bind chemerin [28–30]. It was established that chemerin primarily mediates its functions through CMKLR1 [31]. Besides in adipocytes [21], CMKLR1 has been successively detected in the adaptive immune system including macrophages, natural killer cells, dendritic cells, and leucocytes [24, 28, 32, 33]. Expressed in human endothelial cells, CMKLR1 was activated by TNF-*α*, IL-1*β*, and IL-6. Through this receptor, chemerin activated MAPK and PI3K-AKT signal pathways to promote the proliferation, differentiation, and migration of endothelial cells and induce angiogenesis in vitro [16]. This angiogenesis induced by chemerin can be abolished by the CMKLR1 inhibitor [34]. Acted at CMKLR1, but not GPR1, chemerin promoted VSMC proliferation and increased blood pressure [35, 36]. In the isolated rat thoracic aorta, CMKLR1 agonist chemerin-9 caused arterial contraction in a dose-dependent manner, while CMKLR1 antagonist CCX832 inhibited phenylephrine-induced vasoconstriction [37]. Our previous study indicated that the proliferation and migration of VSMCs induced by platelet-derived growth factor (PDGF) was inhibited by knockdown of CMKLR1 [18]. Our current data suggested that chemerin stimulated the progression of atherosclerosis by promoting p38 MAPK phosphorylation and NF-*κ*Bp65 expression. We did not investigate the role of CMKLR1 in chemerin-mediated plaque formation. Given the evidences of CMKLR1 in vascular biology, it can be speculated that CMKLR1 may play a role in the initiation and progression of atherosclerosis.

In summary, we firstly identified that the circulating and aortic levels of chemerin increased in the progression of aortic atherosclerosis in ApoE^−/−^ mice. Chemerin promoted the initiation and progression of atherosclerosis.

## Data Availability

The data used to support the findings of this study are available from the corresponding author upon request.
